# Sequential Development of Waldenström’s Macroglobulinemia After High-Grade Lymphomatoid Granulomatosis

**DOI:** 10.7759/cureus.80802

**Published:** 2025-03-18

**Authors:** Makoto Ito, Yusei Mizumoto, Yasushi Murakami, Satsuki Nakano, Norio Takagi

**Affiliations:** 1 Department of Hematology, Tokoname City Hospital, Tokoname, JPN; 2 Department of Hematology and Oncology, Graduate School of Medicine, Nagoya University, Nagoya, JPN; 3 Department of Respiratory Medicine, Tokoname City Hospital, Tokoname, JPN; 4 Department of Pathology, Tokoname City Hospital, Tokoname, JPN; 5 Department of Pathology and Molecular Diagnostics, Graduate School of Medical Sciences and Medical School, Nagoya City University, Nagoya, JPN

**Keywords:** bendamustine, lymphomatoid granulomatosis, lymphoplasmacytic lymphoma/waldenström’s macroglobulinemia, rituximab, transbronchial biopsy

## Abstract

Lymphomatoid granulomatosis (LYG) is a rare Epstein-Barr virus-driven B-cell lymphoproliferative disease that often progresses to high-grade lymphoma. We describe a case of high-grade LYG causing Pancoast syndrome, diagnosed via transbronchial biopsy after a failed incisional biopsy. Complete remission was achieved with R-CHOP (rituximab, doxorubicin, cyclophosphamide, vincristine, and prednisolone), but 2.5 years later, the patient developed lymphoplasmacytic lymphoma/Waldenström’s macroglobulinemia (LPL/WM). Despite bendamustine-rituximab improving LPL/WM, LYG recurred, underscoring its treatment challenges. This case highlights LYG’s diagnostic complexity, its potential link with other hematologic malignancies, and therapeutic limitations. Further research is needed to elucidate LYG’s pathogenesis and develop effective treatments for relapsed cases.

## Introduction

Lymphomatoid granulomatosis (LYG) is a rare Epstein-Barr virus (EBV)-driven B-cell lymphoproliferative disease first described by Liebow et al. [1]. LYG is characterized by angiocentric and angiodestructive infiltration of EBV-positive B-cells and is often accompanied by necrosis [2]. The clinical course varies widely, ranging from spontaneous remission to progression into aggressive lymphoma. High-grade LYG has a poor prognosis, with median progression-free survival and overall survival of approximately one and two years, respectively, even with rituximab-based regimens such as R-CHOP (rituximab, doxorubicin, cyclophosphamide, vincristine, and prednisolone) [2,3]. Waldenström’s macroglobulinemia (WM), a subtype of lymphoplasmacytic lymphoma (LPL), is a rare B-cell malignancy characterized by monoclonal IgM production and bone marrow infiltration. The age-adjusted incidence rate of WM/LPL in the United States is approximately 0.63 per 100,000 person-years, with the highest rates observed among White populations (0.74 per 100,000). In Japan, the reported incidence is approximately 0.28 per 100,000 person-years [4]. Even more rarely, LYG is an EBV-associated B-cell lymphoproliferative disorder for which precise epidemiological data are lacking due to its extreme rarity. Although LYG is recognized as a disorder often associated with immunodeficiency states [2], its coexistence or sequential development with LPL/WM has not been reported. The coexistence or sequential development of LPL/WM following other EBV-related lymphoproliferative disorders is extremely rare and, to our knowledge, has not been reported before. In this report, we describe a unique case of LYG that later developed LPL/WM and highlight the diagnostic challenges, disease progression, and therapeutic strategies.

## Case presentation

A 73-year-old woman presented with left chest and arm pain that had persisted for three months and was accompanied by a gradually enlarging palpable tumor in the left neck region. She had no significant medical history, smoking history, or family history of systemic disease or cancer. Physical examination revealed left eyelid ptosis, neck swelling, and tenderness. Laboratory results showed elevated C-reactive protein and soluble interleukin-2 receptor levels but normal immunoglobulin M levels (Table 1). Hyponatremia was likely secondary to the syndrome of inappropriate antidiuretic hormone secretion associated with the tumor or inflammatory cytokine release.

**Table 1:** Complete blood test of the patient at diagnosis

Complete blood test	Observed value	Reference range
White blood cell	9,800/μL	3,300-8,600/μL
Band neutrophil	11.3%	2.0%-13.0%
Segmented neutrophil	57.0%	38.0%-58.9%
Lymphocyte	23.7%	26.2%-46.6%
Monocyte	7.7%	2.3%-7.7%
Basophil	0.3%	0.0%-0.6%
Eosinophil	0.0%	0.0%-1.0%
Red blood cell	446 × 10^4^/μL	410-510 × 10^4^/μL
Reticulocyte	12‰	2‰-17‰
Hemoglobin	11.9 g/dL	13.7-16.8 g/dL
Platelet	27.2 × 10^4^/μL	15.8-34.8 × 10^4^/μL
Albumin	3.7 g/dL	4.1-5.1 g/dL
Total bilirubin	0.4 mg/dL	0.2-1.0 mg/dL
Aspartate aminotransferase	28 U/L	13-30 U/L
Alanine aminotransferase	12 U/L	8-20 U/L
Lactate dehydrogenase	290 U/L	124-222 U/L
Blood urea nitrogen	8.8 mg/dL	8.0-20.0 mg/dL
Creatinine	0.65 mg/dL	0.65-1.07 mg/dL
Sodium	131 mEq/L	138-145 mEq/L
Potassium	4.3 mEq/L	3.6-4.8 mEq/L
Chloride	95 mEq/L	101-108 mEq/L
Calcium	9.5 mg/dL	8.8-10.1 mg/dL
Ferritin	632.1 ng/mL	40-188 ng/mL
C-reactive protein	6.20 mg/dL	0.00-0.14 mg/dL
Carcinoembryonic antigen	0.79 ng/mL	0.0-5.0 ng/mL
Cytokeratin 19 fragment	0.0 ng/mL	0.0-3.5 ng/mL
Pro-gastrin-releasing peptide	29.8 pg/mL	<80 pg/mL
Soluble interleukin-2 receptor	2,140 U/mL	145-519 U/mL
Immunoglobulin G	924 mg/dL	870-1,700 mg/dL
Immunoglobulin A	162 mg/dL	110-410 mg/dL
Immunoglobulin M	49 mg/dL	33-190 mg/dL
Epstein-Barr virus-viral capsid antigen-immunoglobulin M	<10	<10
Epstein-Barr virus-viral capsid antigen-immunoglobulin G	1:40	<10
Epstein-Barr virus nuclear antigen	1:10	<10
Human immunodeficiency virus antibody	Negative	Negative

**Figure 1:**
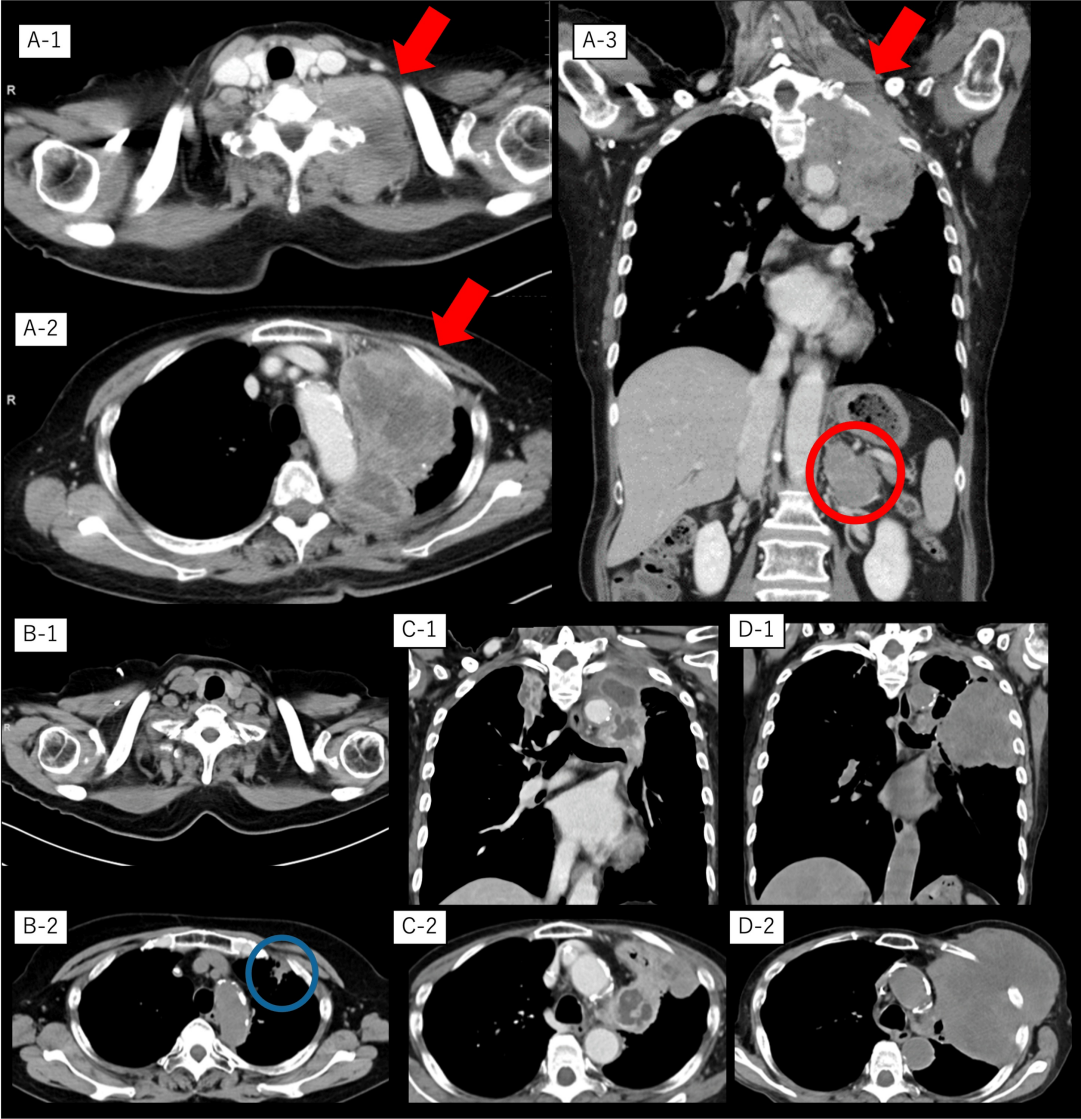
Computed tomography images of the patient during the clinical course Contrast-enhanced computed tomography images at hospitalization (A-1, A-2, and A-3), after six cycles of R-CHOP (rituximab, doxorubicin, cyclophosphamide, vincristine, and prednisolone) therapy (B-1 and B-2), at relapse of lymphomatoid granulomatosis (C-1 and C-2), and just before the patient passed away (D-1 and D-2). Red arrows indicate the primary lesion from the left lung apex to the neck; the red circle indicates a metastatic lesion in the left adrenal gland. A transbronchial lung biopsy performed on the residual lesions showed organizing pneumonia (blue circle).

**Figure 2:**
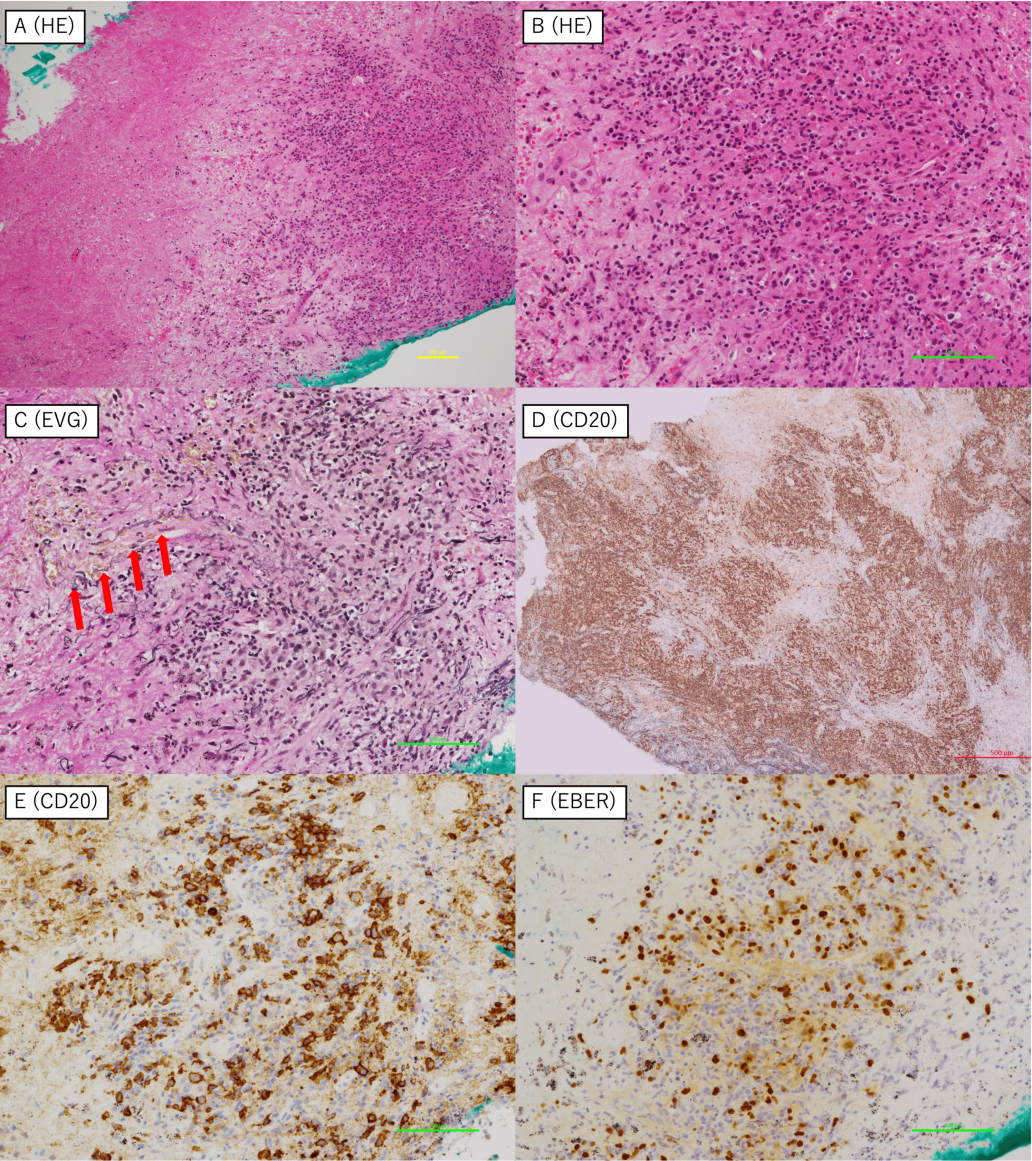
Histopathological findings of the lung specimens at the diagnosis of lymphomatoid granulomatosis Histopathological examination revealed prominent coagulative necrosis (A) and angiocentric and angiodestructive lymphoid infiltration (B, C). Red arrows indicate the destructed vessels on Elastica van Gieson (EVG) staining (C). CD20-positive atypical large B-cells infiltrated in clusters (D, E), and these lymphocytes were positive for Epstein-Barr virus-encoded RNA (EBER) (F). HE: hematoxylin-eosin

**Figure 3:**
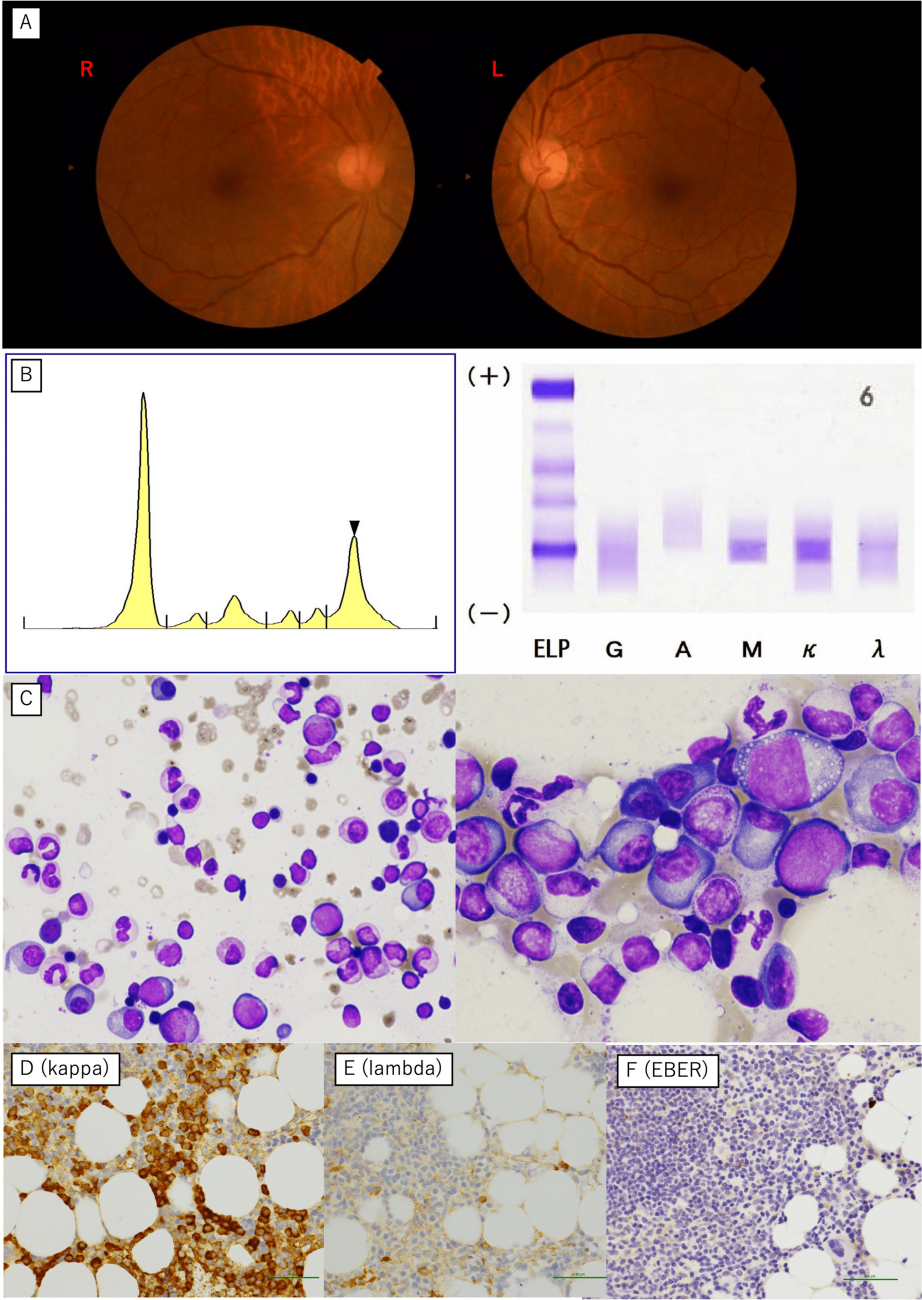
Findings observed at the diagnosis of lymphoplasmacytic lymphoma/Waldenström’s macroglobulinemia Marked venous engorgement (sausaging) was observed (A). Serum protein electrophoresis and immunoelectrophoresis revealed M-peak (arrowhead) and IgM-kappa type M-protein (B). May-Giemsa staining showed an increased number of small lymphocytes and plasma cells in the bone marrow (C). Immunohistochemical staining revealed positive for kappa light chains (D) but negative for lambda light chains (E) and Epstein-Barr virus-encoded RNA (EBER) (F).

Contrast-enhanced computed tomography (CT) revealed a 9-cm mass extending from the left pulmonary apex to the neck, along with left adrenal metastasis as shown in Figure 1A-1 to Figure 1A-3.

Although a transbronchial biopsy (TBB) was rapidly performed, it yielded seemingly insufficient diagnostic material due to prominent necrosis observed on hematoxylin-eosin staining. An incisional biopsy of the supraclavicular lesion was also performed to obtain a certain amount of tumor specimens. While the additional biopsy confirmed only large B-cell lymphoma, TBB ultimately established a diagnosis of grade 3 LYG through histopathological findings, including angiodestruction, angioinvasion, coagulative necrosis, and EBV-encoded RNA (EBER) positivity (Figure 2).

Given the diagnosis of high-grade LYG with Pancoast syndrome (Horner’s syndrome and brachial plexus involvement), R-CHOP therapy was initiated immediately. After one cycle, the patient experienced rapid pain relief, obviating the need for analgesics. Following six R-CHOP cycles and three cycles of additional rituximab monotherapy, complete remission (CR) was achieved. Follow-up CT performed one year after the initial chemotherapy revealed a suspected residual lesion in the left lung apex. Subsequent TBB of this area led to a diagnosis of organizing pneumonia, suggesting that the lesion represented post-chemotherapy scarring rather than residual malignancy as shown in Figures 1B-1, 1B-2.

However, 2.5 years later, the patient developed retinopathy due to hyperviscosity, a rapidly elevated IgM level (5,534 mg/dL, reference range: 33-190 mg/dL), and thrombocytopenia (platelet count: 24,000/μL, reference range: 15.8-34.8 × 104/μL). Bone marrow biopsy confirmed LPL/WM, which was characterized by monoclonal IgM-kappa production and EBER negativity (Figure 3). The markedly elevated IgM level was indicative of a hyperviscosity syndrome, explaining the development of retinopathy. Thrombocytopenia likely resulted from bone marrow infiltration by lymphoplasmacytic cells. The detection of monoclonal IgM-kappa protein confirmed the diagnosis of WM. EBER negativity in the bone marrow further supported the notion that the newly diagnosed lymphoma was unrelated to prior EBV-driven LYG.

**Figure 4:**
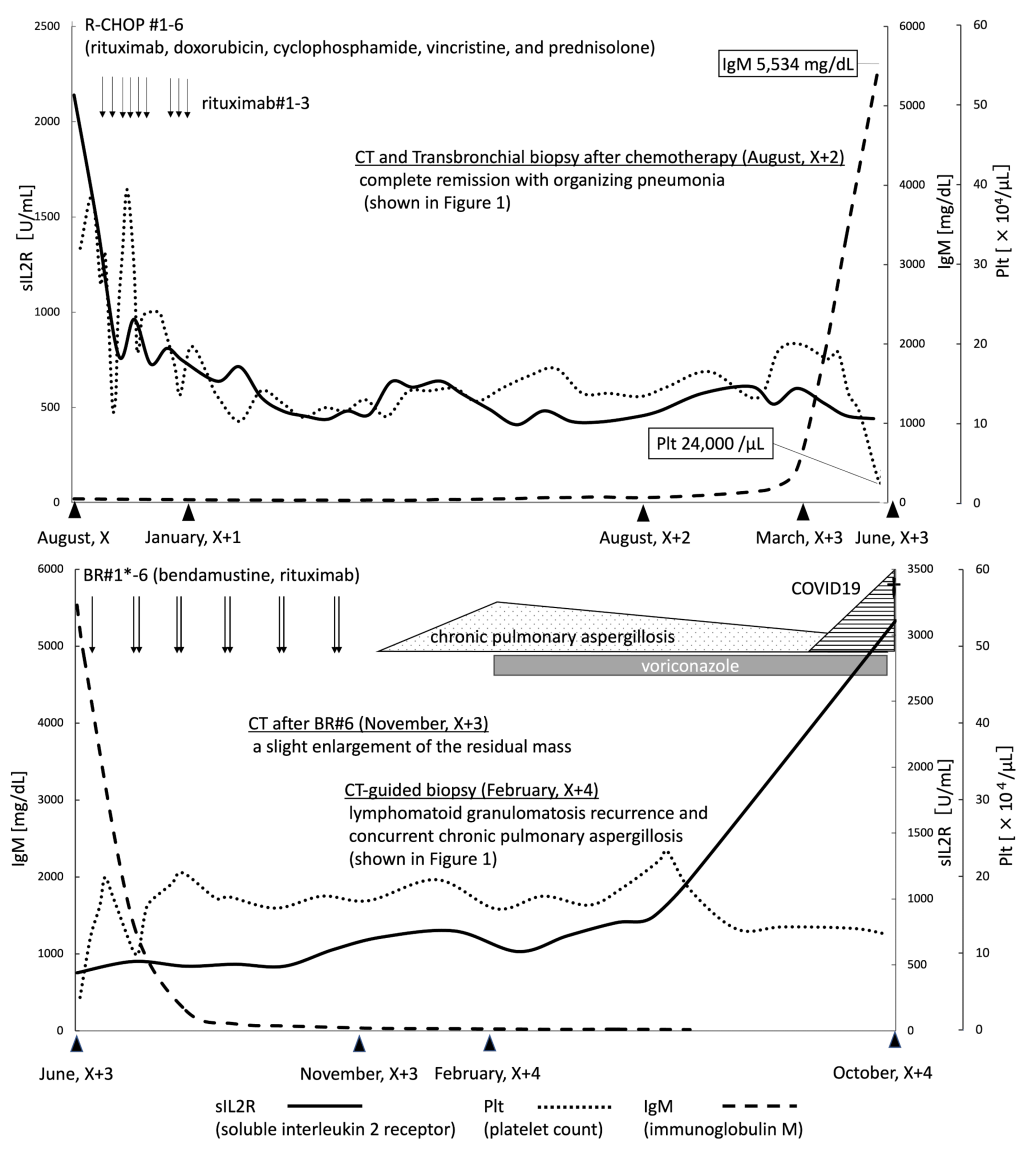
The overall clinical course of the patient The patient visited our hospital in August of year X, developed Waldenström’s macroglobulinemia in March of year X+3, and passed away in October of year X+4. *We administered bendamustine monotherapy during the first course of BR therapy. BR: bendamustine-rituximab; CT: computed tomography

At the time of WM diagnosis, when LYG was still in remission, six cycles of bendamustine-rituximab (BR) were administered for LPL/WM, resulting in the clinical improvement of retinopathy, IgM levels, and platelet counts. However, follow-up CT after six cycles of BR revealed progressive thoracic mass enlargement and internal fluid retention. CT-guided biopsy confirmed LYG recurrence with concurrent *Aspergillus fumigatus* infection as shown in Figures 1C-1, 1C-2. The patient declined further chemotherapy, opting for the best supportive care. Despite antifungal treatment with voriconazole, the patient succumbed to COVID-19-related complications. The entire treatment trajectory is shown in Figure 4.

## Discussion

To our knowledge, this is the first reported case of WM developing after high-grade LYG, highlighting a potentially underrecognized association between these rare B-cell malignancies. This case underscores three critical aspects of LYG and its management: diagnostic challenges, secondary hematologic malignancies, and therapeutic strategies.

Diagnostic challenges

The diagnosis of LYG is inherently challenging because the disease is exceedingly rare and exhibits very subtle histological features [5]. The initial differential diagnoses in this case included lung cancer, granulomatosis with polyangiitis, other EBV-associated lymphoproliferative disorders, and infectious causes. However, histopathological findings such as angiocentric and angiodestructive infiltration, necrosis, and EBER positivity in B-cells by in situ hybridization were highly characteristic of LYG and allowed us to distinguish it from these other conditions. TBB has emerged as a less invasive alternative to surgical lung biopsy in lung cancer diagnosis, although its diagnostic yield and sensitivity are slightly lower than those of surgical biopsy [6]. TBB may occasionally fail to provide an accurate diagnosis due to tissue contusion caused by mechanical clamping, necessitating subsequent surgical biopsy. Nevertheless, the TBB specimen in this case was analyzed successfully and presented the features of LYG grade 3, including angiocentric infiltration, coagulative necrosis, and EBER positivity. This observation is interesting, as lesions at the leading edge of the tumor may not consistently exhibit the hallmark histopathological findings of LYG. Although the exact mechanism underlying this phenomenon remains unclear, these findings emphasize the importance of the multidimensional evaluation of LYG. The detection of EBER positivity via TBB highlighted its diagnostic value even when the incisional biopsy is inconclusive. Furthermore, transbronchial lung cryobiopsies can provide larger, higher-quality tissue samples [7], which may enhance the LYG diagnosis.

Cooccurrence of hematologic malignancies

This is the first reported case of WM developing after high-grade LYG, highlighting a potentially underrecognized association between these rare B-cell malignancies. Previous reports have described the development of LYG in patients with immunodeficiency disorders, such as Wiskott-Aldrich syndrome and acquired immunodeficiency syndrome [8,9]. Several LYG cases also developed secondary to multiple myeloma, T-cell large granular lymphocytic leukemia, and chronic lymphocytic leukemia [10-13]. These findings demonstrate that the pathogenesis arises from defective immune surveillance. To better highlight the rarity and clinical significance of our case, previously reported cases of LYG associated with other hematologic malignancies have been summarized in Table 2.

**Table 2:** Cases of lymphomatoid granulomatosis and lymphoplasmacytic lymphoma/Waldenström’s macroglobulinemia related to other hematologic malignancy LYG: lymphomatoid granulomatosis; LPL/WM: lymphoplasmacytic lymphoma/Waldenström’s macroglobulinemia

Case	Authors	Journal	Year	Associated hematologic malignancy	Timing of onset	Summary
LYG with hematologic malignancy cases						
1	Ragage et al.	Ann Pathol	2006	Acute myeloid leukemia (AML)	Prior to LYG	Case of pulmonary LYG developed in an immunocompromised state after AML treatment [14]
2	Kim	Korean J Spine	2012	Acute lymphoblastic leukemia (ALL)	Prior to LYG	Spinal LYG after treatment for childhood ALL [15]
3	Michot et al.	Leuk Lymphoma	2013	T-large granular lymphocyte leukemia (T-LGL)	Prior to LYG	T-LGL preceded the diagnosis of LYG by 3 years, suggesting that LG is secondary to T-LGL [12]
4	De Luca et al.	Leuk Lymphoma	2018	T-LGL	Prior to LYG	Pulmonary LYG occurred after T-LGL leukemia development [11]
5	Rezvani et al.	Case Rep Pulmonol	2019	Chronic lymphocytic leukemia (CLL)	Prior to LYG	Report of rapidly progressive pulmonary LYG in a patient with CLL [13]
6	Lee et al.	Intern Med	2019	Multiple myeloma (MM)	Simultaneous	Simultaneous onset of CNS/pulmonary LYG and MM under immunosuppressive conditions [10]
7	Szczepanek et al.	Front Neurol	2020	CNS Hodgkin lymphoma (HL)	Simultaneous	Simultaneous onset of CNS HL and pulmonary LYG during immunosuppressive therapy for ulcerative colitis [16]
8	Bi et al.	Front Oncol	2022	Chronic myeloid leukemia (CML)	Prior to LYG	CML patient developed LYG and pulmonary infection during treatment [17]
LPL/WM after B-cell malignancy cases						
9	Uğur et al.	Tepecik Eğit Hast Derg	2014	Non-Hodgkin lymphoma (NHL)	WM after NHL treatment	WM occurred 3 years after NHL treatment; reported as secondary malignancy [18]
10	Samsuddoha et al.	Cureus	2024	Chronic lymphocytic leukemia (CLL)	WM after CLL	Very rare case of CLL transforming into WM during ibrutinib therapy [19]
LYG-WM/LPL association case						
11	Melegh et al.	Pathol Res Pract	2009	Lymphoplasmacytic lymphoma (LPL)	LYG after LPL treatment	Cervical LYG occurred under immunosuppression after treatment for LPL [20]

This comparison highlights the unique sequence and diagnostic complexity of the present case. In the present case, however, immunodeficiency in the patient was not evident. Although the risk of developing LPL/WM increases after developing LYG or administering immunochemotherapy remains unknown [4], two hypotheses were considered: (1) a shared tumorigenic origin and (2) LPL/WM emergence secondary to immunosuppression. Although the absence of elevated IgM levels and EBER positivity at LYG onset might be evidentiary against a shared etiology, favoring the latter hypothesis, we could not perform genetic analysis to support this hypothesis. This highlights a major limitation of this study, indicating the need for further molecular studies to elucidate the mechanisms underlying this lymphomagenesis.

Therapeutic challenges

While the patient achieved CR after R-CHOP, BR was ineffective against early-stage recurrent LYG despite demonstrating some efficacy against LPL/WM. Recent evidence indicates that dose-adjusted-EPOCH-R (etoposide, prednisone, vincristine, cyclophosphamide, doxorubicin, and rituximab) is a promising first-line therapy for high-grade LYG [21]. However, an effective regimen has not yet been established for refractory/relapsed cases. To date, only two cases of LYG treated with bendamustine have been documented. However, these cases were treated by brentuximab-vedotin combination therapy. No cases treated with BR therapy have been reported [22,23]. These studies indicate that unregulated immune control of EBV may contribute to disease relapse after chemotherapy, signifying the critical need for novel immunotherapies, including programmed cell death protein-1 blockade. The results of the ongoing prospective study will be of interest (Clinical Trials number: NCT03258567).

## Conclusions

This case illustrates an exceptionally rare clinical trajectory in which LPL/WM developed sequentially after high-grade LYG. The diagnostic complexity presented in this case due to overlapping clinical features, evolving histopathological findings, and limitations in biopsy sampling emphasizes the importance of comprehensive and multidimensional diagnostic approaches. Furthermore, the treatment course highlights the therapeutic challenges in managing relapsed LYG, for which standardized regimens are lacking. Although the coexistence or sequential development of these rare B-cell malignancies is extremely uncommon, this case underscores the importance of long-term surveillance and consideration of secondary hematologic malignancies in patients with EBV-associated lymphoproliferative disorders. Future research is warranted to elucidate the underlying pathogenesis and to develop more effective therapeutic strategies.
